# Host genetic variation at a locus near CHD1L impacts HIV sequence diversity in a South African population

**DOI:** 10.1128/jvi.00954-23

**Published:** 2023-09-25

**Authors:** Vanessa E. Schulz, Jeffrey F. Tuff, Riley H. Tough, Lara Lewis, Benjamin Chimukangara, Nigel Garrett, Quarraisha Abdool Karim, Salim S. Abdool Karim, Lyle R. McKinnon, Ayesha B. M. Kharsany, Paul J. McLaren

**Affiliations:** 1 Department of Medical Microbiology and Infectious Diseases, University of Manitoba, Winnipeg, Manitoba, Canada; 2 Sexually Transmitted and Bloodborne Infections Division, JC Wilt Infectious Diseases Research Centre, National Microbiology Laboratories, Public Health Agency of Canada, Winnipeg, Manitoba, Canada; 3 Centre for the AIDS Programme of Research in South Africa, Durban, South Africa; 4 Critical Care Medicine Department, NIH Clinical Center, National Institutes of Health, Bethesda, Maryland, USA; 5 Department of Virology, University of KwaZulu-Natal, Durban, South Africa; 6 Department of Public Health Medicine, School of Nursing and Public Health, University of KwaZulu-Natal, Durban, South Africa; 7 Department of Epidemiology, Mailman School of Public Health, Columbia University, New York, New York, USA; 8 Department of Medical Microbiology, School of Laboratory Medicine and Medical Science, Nelson R. Mandela School of Medicine, University of KwaZulu-Natal, Durban, South Africa; Ulm University Medical Center, Ulm, Baden-Württemberg, Germany

**Keywords:** HIV, genome-to-genome analysis, host-pathogen interaction, viral genomics, host genomics, HLA, viral load

## Abstract

There is variability in viral load (VL) among individuals with untreated human immunodeficiency virus (HIV) infection, and this variability can be partly attributed to host genetics. HIV is known to develop escape mutations to evade host immune pressure, particularly from HLA alleles and, in some cases, counteracts the protective effect of host alleles. A recent genome-wide association study (GWAS) of HIV VL in individuals of African ancestry identified a locus on chromosome 1, near the protein-coding gene chromodomain helicase DNA-binding protein 1 like (*CHD1L*), that has a novel association with control of HIV replication. However, not all individuals carrying the protective alleles maintain low VL, and the region’s impact on viral evolution has not been investigated. To address this, we conducted a host-virus regional association analysis in 147 people living with HIV (PLWH) from South Africa with both human and viral genome data available. We observed significant associations between the *CHD1L* variants rs77029719 (G) (*P* = 1.6 × 10^−2^), rs7519713 (T) (*P* = 2.3 × 10^−2^), and rs59784663 (G) and 73004025 (T) (*P* = 1.4 × 10^−2^) with codon 248 of HIV reverse transcriptase (RT) and between *CHD1L* variant rs7519713 (T) and codon 18 (*P* = 3.2 × 10^−2^) and 147 (*P* = 3.9 × 10^−2^) of HIV gag. These associations are consistent with viral escape from CHD1L pressure. In addition, we observed significant associations between HLA B*81 (*P* = 1.5 × 10^−5^) and HLA C*18 (*P* = 7.0 × 10^−4^) with RT codon 4 and HLAB*58 with RT codon 196 (*P* = 9.0 × 10^−4^). This study reveals new evidence of host genetic variation impacting viral evolution in a population highly affected by HIV.

**IMPORTANCE:**

It has been previously shown that genetic variants near *CHD1L* on chromosome 1 are associated with reduced HIV VL in African populations. However, the impact of these variants on viral diversity and how they restrict viral replication are unknown. We report on a regional association analysis in a South African population and show evidence of selective pressure by variants near *CHD1L* on HIV RT and gag. Our findings provide further insight into how genetic variability at this locus contributes to host control of HIV in a South African population.

## INTRODUCTION

No cure for HIV has been ascertained in the 40 years since its discovery, and it remains a major public health concern that disproportionately affects people living in low-income countries (1). HIV disease progression is variable among untreated individuals and their viral load (VL), measured as HIV RNA copies/mL of plasma, is a predictor of disease progression (2 – 5) and transmission potential (6 – 9). Several factors contribute to an individual’s VL during infection including their environment, gender/sex, use of antiretroviral treatment (ART), attributes of the infecting virus, co-morbidities (including co-infections), and host genetics (2 – 4, 10, 11). Host genetic studies, primarily in populations of European ancestry, have consistently shown associations between variation in *HLA* and *CCR5* with VL, accounting for up to 25% of VL variability in people not receiving ART (4, 12). In addition to host genetics, HIV sequence variation contributes ~30% to variability in VL (4, 5, 11, 12).

A key component among individuals with untreated HIV infection is within-host viral evolution, where the virus adapts to its environment to maximize replication and transmission potential (13, 14). The best evidence for viral adaptation to host genetic variation comes from numerous studies identifying viral escape mutations from *HLA* class I alleles (13, 15 – 19). For example, HLAB*51 restricts an epitope in reverse transcriptase (RT) which selects for the I135X escape mutation in ~96% of HLAB*51 carriers and interferes with CD8^+^ T-cell recognition (13, 18). Importantly, HLAB*51 was protective against HIV disease progression prior to 1997 in Japanese populations where the allele frequency is high, but is no longer protective due to the accumulation of I135x within the population (13). Another example is HLAB*57:03, which is known to reduce VL and drives the T242N mutation in the gag TW10 epitope in ~80% of HLAB*57:03 carriers (17, 18, 20 – 22). In contrast to HLAB*51, the T242N mutation has a viral fitness cost, and individuals carrying the protective allele exhibit slow disease progression (13, 17).

In a genome-wide screen comparing host genetic variation to viral genetic variation, in ~1,000 individuals of European ancestry, Bartha et al. observed evidence of an association between *HLA* alleles and amino acid (AA) variants across the HIV proteome (14). Notably, the associations between *HLA* alleles and viral variation were many orders of magnitude stronger than those observed between VL and *HLA* alleles. These findings suggest that the viral genome may provide more power to detect signatures of host pressure on the virus, compared to clinical features.

One major shortcoming of current HIV host genomic studies is that they have largely been performed in individuals of European ancestry with clade B HIV infection. Therefore, these studies do not interrogate the majority of human and HIV genetic diversity or the populations most impacted by HIV. In a GWAS of 3,879 individuals of African ancestry, the International Collaboration for the Genomics of HIV (23) identified a locus on chromosome 1, near the protein-coding gene *CHD1L*, that is associated with reduced VL (24). The top associated variant in this region, rs59784663 (G), is associated with a ~0.3 log_10_ copies/mL reduction in VL and is only present at high frequency in individuals of African ancestry. Additional analyses using a two-way analysis of variance found that two single nucleotide polymorphisms (SNPs), rs73004025 and rs7519713, in high linkage disequilibrium with the top variant, had a larger combined impact on VL than rs59784663 (G) alone. However, ~18% of individuals who carry the protective alleles at this locus still exhibit high VLs, defined as being in the top quartile of the VL distribution, suggesting the protective effect is not absolute.

**FIG 1:**
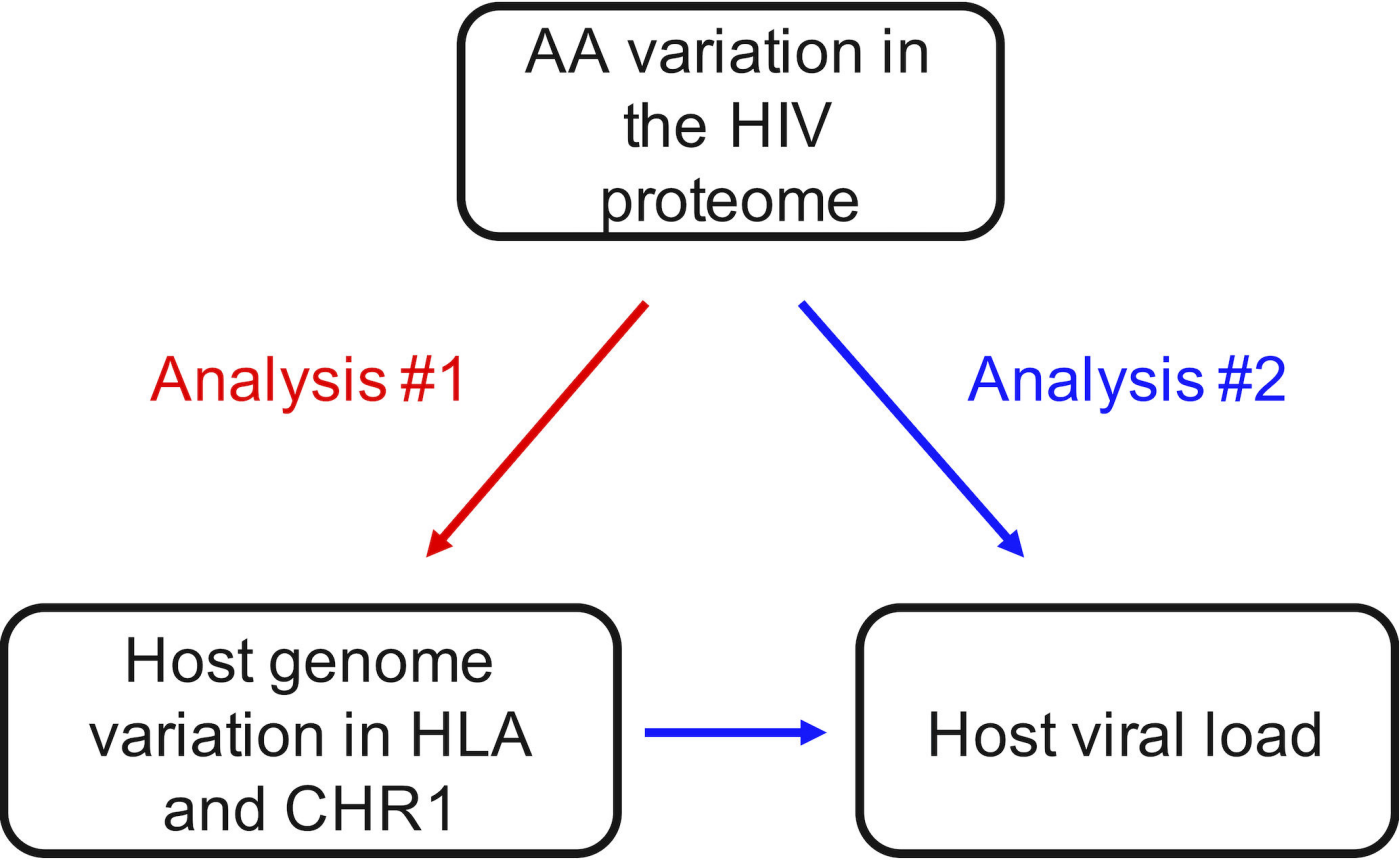
The two analyses done in the present study to investigate associations between the host and viral genome and viral load. AA, amino acid; CHR, chromosome.

Here, we hypothesize that HIV mutates to counteract the protective effect of chromosome 1 variants, just as it does to abrogate the protection afforded by certain *HLA* alleles. We tested this hypothesis by conducting a regional association analysis on 147 PLWH from South Africa and assessed the impact of host genetic variation in the chromosome 1 and *HLA* regions on viral sequence diversity and VL (Fig. 1). We also investigated predicted epitope binding affinities for significant *HLA*-to-viral variant associations. We demonstrate that viral sequence variation can serve as a phenotype for association analyses and identify areas of the viral genome that associate with host genetic variants that impact VL. This study further investigates host-HIV interaction in an understudied population, increasing our understanding of viral evolution and host control of HIV.

## MATERIALS AND METHODS

### Study populations

We obtained host and viral genetic data from two cohort studies. The HIPSS study was a community-wide study undertaken in KwaZulu-Natal, South Africa, between the years of 2014 and 2017 to determine the association of contemporaneous programmatic scale-up of prevention and treatment efforts on HIV prevalence and incidence (25). Genotyping information for 97 participants of the HIPSS (25) with available HIV sequence data was acquired through collaboration with the University of KwaZulu-Natal, South Africa. HIPSS participants selected for this study were PLWH, had CD4^+^ T-cell counts of >350 cells/mm^3^ of plasma, and self-reported as ART naïve. The HIPSS cohort comprised men and women from urban and rural communities. For the VL data, it is unknown at which point in the individuals’ infection course the VL measurement was taken.

The CAPRISA-004 trial was a randomized placebo-controlled trial undertaken in KwaZulu-Natal, South Africa, from 2007 to 2010 to assess the safety and effectiveness of 1% tenofovir gel for the prevention of HIV infection in women (26). Women who seroconverted were referred to the CAPRISA AIDS Treatment Program and offered enrollment into the CAPRISA-002 Acute Infection Study (27). We acquired genotyping information for 71 PLWH from the CAPRISA-004 trial (26), with or without prior exposure to tenofovir gel, of which we had access to clinical data for 53 individuals who self-reported as ART naïve in the first year of infection. VL was measured 1 year post-seroconversion, and individuals with VL data below the limit of detection (<400 copies/mL of plasma) were assigned a log_10_ VL of 1.3. Previously collected plasma samples were used for viral sequencing (26).

The HIPSS study comprised 20,048 total participants (25), and the CAPRISA-004 study comprised 889 total participants. Thus, the genotyping data for the 97 and 71 selected individuals constituted a small percentage of the study populations.

### Host genotype data

**FIG 2:**
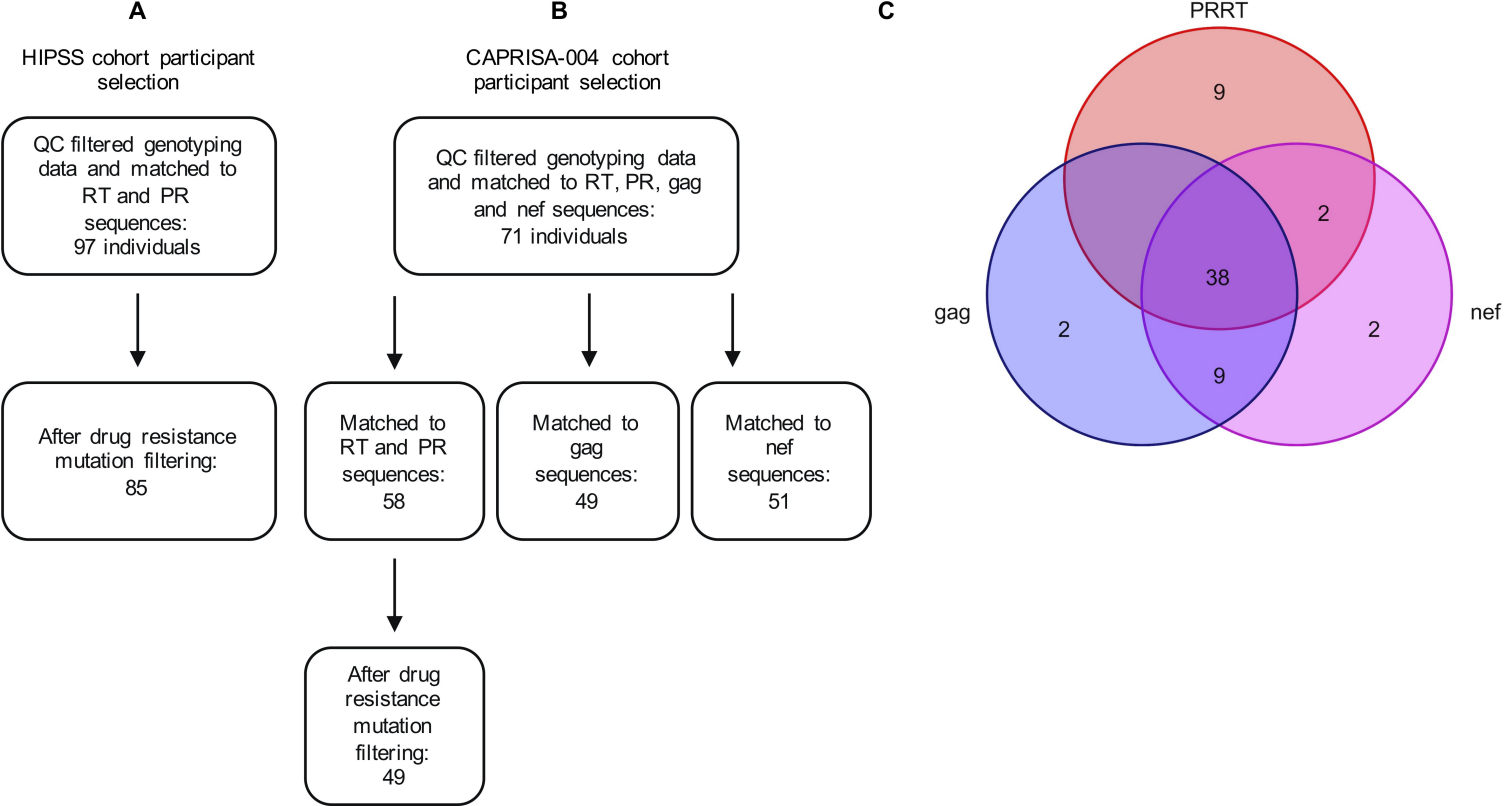
(**A**) HIPSS and (**B**) CAPRISA-004 cohort filtering. (**C**) The number of individuals that have sequences shared across the different regions of the HIV genome for CAPRISA. PR, protease; PRRT, protease and reverse transcriptase; QC, quality control; RT, reverse transcriptase.

Host genotyping was done using the custom H3Africa microarray (Illumina). The microarray consisted of 2,267,346 tag SNPs enriched with genetic content to improve resolution of genomic diversity in African populations. Quality control (QC) filtering was done with PLINK version 1.9 (28 – 31) using a previously described protocol (32). Study participants had been filtered using thresholds of missingness of >2%, heterozygosity rate of >3 standard deviations from the mean, and cryptic relatedness of >0.2. SNPs were filtered using thresholds of missingness of >2%, minor allele frequency (MAF) of <0.01, and Hardy-Weinberg equilibrium of *P* > 1×10^−6^. Principal component analyses were done using PLINK v1.9. *RT,* and protease (*PR*) sequences were matched to 97 HIPSS and 58 CAPRISA-004 individuals with high-quality genomic data (Fig. 2A). *Gag* and *nef* sequences were matched to CAPRISA-004 individuals, resulting in 49 and 51 individuals, respectively (Fig. 2B and C).

### Viral sequence data

For *RT* and *PR*, we obtained 97 HIPSS and 58 CAPRISA-004 sequences for 99 codons of the *PR* gene and the first 255 codons of the *RT* gene (26, 33). HIV sequences for CAPRISA-004 were generated after seroconversion was identified (26). For HIPSS, it is unknown at which point in the individuals’ infection course the virus was sequenced. Sequences were generated via Sanger sequencing using ABI 3130, and consensus sequences were aligned with Sequencher version 4.5 (26) or Clustal W (33). *RT* and *PR* sequences were run through the Stanford University HIV Drug Resistance Database version 9.1 (https://hivdb.stanford.edu/hivdb/by-sequences/) to identify individuals with drug resistance mutations, viral subtype, and for AA alignment. Twelve HIPSS and nine CAPRISA-004 individuals were removed due to the presence of drug resistance mutations. This left 85 HIPSS and 49 CAPRISA-004 individuals for analysis (Fig. 2A, B, and C). All 85 HIPSS viral sequences were subtype C; 48 CAPRISA-004 sequences were subtype C; and 1 CAPRISA-004 sequence was subtype A/D. AA codons with ≥10% and ≤90% of individuals with a non-consensus AA were selected for statistical testing. A variant AA was described as an AA that differed from the consensus sequence. Empty codons added to accommodate the alignment were considered to have no information for that position. After filtering, there were up to 15 PR codons and 22 RT codons left for statistical testing.

We also obtained 49 sequences from the CAPRISA-004 cohort for 501 codons covering the *gag* gene and 51 sequences for 207 codons covering the *nef* gene (Fig. 2B and C) (34, 35) (GenBank accession numbers: EU347404-EU347714, KF208740-KF208816, and KF208817-KF208898). *Gag* and *nef* sequences were generated from plasma samples within 6 months from seroconversion (35). Sequences were previously subtyped, and all but one sequence for *gag* and *nef* was subtype C, with the other being related to subtype A/D. Viral sequences were aligned with default settings and translated into peptide sequences using the Gene Cutter online tool from the Los Alamos HIV sequence database (https://www.hiv.lanl.gov/content/sequence/GENE_CUTTER/cutter.html). Codons for statistical testing were selected using the protocol described above. After filtering, there were 93 gag codons and 50 nef codons left for statistical testing.

### Chromosome 1 and HLA imputation

To obtain information on classical *HLA* alleles and SNPs in the chromosome 1 region not directly genotyped, we performed genotype imputation in two ways. For chromosome 1, TOPMed (36) imputation was done using Minimac version 4.0 (37), EAGLE, version 2.4 phasing, and GRCh38-hg38 array build. *HLA* imputation was done using the Michigan Imputation Server (36) with Minimac version 4.0 (37), a multi-ethnic *HLA* reference panel, GRCh37/hg19 array build and EAGLE version 2.4 phasing. This approach has been demonstrated to produce highly accurate *HLA* allele information in multiple different ancestry groups, including African individuals (38). Imputed SNPs and *HLA* alleles were filtered using thresholds of MAF of <0.05 and *R* ^2^ of <0.6. The four protective chromosome 1 SNPs (rs59784663, rs73004025, rs77029719, and rs7519713) and 22 two-digit resolution *HLA* alleles were selected from the filtered imputed data set. Rs73004025 was typed in the HIPSS cohort, and the other three SNPs were imputed (*R* ^2^ >0.98). All four SNPs were imputed for CAPRISA (*R* ^2^ >0.97). All 22 *HLA* alleles were imputed for both cohorts (*R* ^2^ >0.63).

### Host genome-to-viral genome association testing

Analysis 1 (Fig. 1) involved testing the association between host SNPs and viral AA variants. Logistic regression was done in PLINK v1.9 using a binary phenotype of consensus or variant AAs. The first principal component was used as a covariate to account for population structure. Association testing was initially completed with HIPSS as a discovery cohort and the CAPRISA-004 cohort for replication. The significance threshold for the *HLA* alleles was adjusted by Bonferroni correction per analysis based on the number of alleles observed (*P* < 2.3 × 10^−3^ for 22 alleles). For chromosome 1 SNPs, the significance threshold was set at *P* < 4.0 × 10^−2^, reflecting the high linkage disequilibrium between the variants (*R* ^2^ >0.8). False discovery rate (FDR) was also calculated for all *P* values from a single analysis (i.e., the *P* values from the association of the *n*th protein codon with the 4 chromosome 1 SNPs and 22 *HLA* alleles). The FDR is based on the distribution of *P* values, accounts for the number of *P* values input, and is calculated as


$$FDR=\;\frac{\pi_{0}\;m\;p_{n}}{n_{pn\;}\leq \;p_{n}}$$

where *m* is the total number of statistical tests; *λ* is the threshold for truly null statistical tests (set at 0.5); *p*_*n*_ is the *P* value of the *n*th statistical test; and *n*_pn_ is the number of *P* values that are ≤ or ≥ the specified variable. For FDR, *π*_0_ is calculated as


$$\pi_{0}=\;\frac{n_{pn}\;\geq \;\lambda }{m\;\left(1-\;\lambda \right)}$$

To summarize, 85 HIPSS and 62 CAPRISA-004 individuals were selected for analyses. Up to 15 PR, up to 22 RT, 93 gag*,* and 50 nef codons were selected for statistical testing with 22 two-digit classical class I *HLA* alleles and 4 SNPs on chromosome 1.

### Epitope predictions and allele dosage association testing

Epitope predictions for significant *HLA*-to-viral codon associations were done using TepiTool (39). Epitopes 15 AAs in length covering the codon of interest were provided to the program, and the associated *HLA* allele was selected for a high number of peptides using TheImmune Epitope Database (IEDB) recommended prediction method. The recommended significance threshold for selecting potential binders is % rank of <1 and IC50 <500 nM (40). Analysis 2 (Fig. 1) investigated the association of host allele dosage-to-VL and variant viral AA-to-VL using a two-sample *t*-test. *t*-Tests and box plots were done in RStudio version 1.3.1056 (41). The significance threshold was set at *P* < 0.05.

## RESULTS

### Chromosome 1 SNPs are significantly associated with variant amino acids in reverse transcriptase

We first conducted a regional association study to test whether *HLA* alleles and chromosome 1 variants significantly associate with AA changes in the HIV proteome. Variants in individuals from the HIPSS cohort were tested for association with AA variants in 7 PR and 20 RT codons. We observed significant associations between host alleles and AA variants in RT (Table 1) but not in PR (Table S2). Specifically, significant associations were found between chromosome 1 variants rs59784663 (G)/rs73004025 (T) (*P* = 1.4 × 10^−2^; OR = 4.9, 95% CI = 1.4–17.5), rs77029719 (G) (*P* = 1.6 × 10^−2^; OR = 4.8, 95% CI = 1.3–17.0), and rs7519713 (T) (*P* = 2.3 × 10^−2^; OR = 4.3, 95% CI = 1.2–15.2) with RT codon 248. In addition, the HIPSS cohort had significant associations between HLA B*81 (*P* = 1.5 × 10^−5^; OR = 39.8, 95% CI = 7.5–211.4) and HLA C*18 (*P* = 7.0 × 10^−4^; OR = 12.5, 95% CI = 2.9–54.2) with RT codon 4 (epitope SL10), and HLAB*58 with RT codon 196 (*P* = 9.0 × 10^−4^; OR = 9.1, 95% CI = 2.5–33.8). The odds ratios show that variant AAs in these codons of RT are observed more in individuals with these significant alleles, with HLAB*81 having a particularly strong association with RT codon 4. In order to test the reproducibility of the HIPSS associations, we next performed a similar analysis in the CAPRISA cohort (*n* = 49). A combined analysis of both cohorts together did not uncover any additional associations, and we did not observe any significant associations in the CAPRISA cohort at the sites implicated in the HIPSS cohort (Table 1) or any other sites in PR and RT (Tables S5 and S6).

**TABLE 1:** Associations between HIPSS host alleles and viral AA for HIV RT (*n* = 85)^*b*^

Host allele	Codon	AA	HIPSS *P* value	FDR	OR	95% CI	Joint *P* value	FDR	OR	95% CI
rs59784663/rs73004025	248	E248D	1.4 × 10^−2^ ^*a*^	2.6 × 10^−1^	4.9	1.4–17.5	9.6 × 10^−2^	3.1 × 10^−1^	2.5	0.8–7.7
rs77029719	248	E248D	1.6 × 10^−2^ ^*a*^	9.4 × 10^−2^	4.8	1.3–17.0	2.1 × 10^−1^	3.7 × 10^−1^	1.9	0.7–5.5
rs7519713	248	E248D	2.3 × 10^−2^ ^*a*^	1.0 × 10^−1^	4.3	1.2–15.2	2.8 × 10^−1^	4.0 × 10^−1^	1.8	0.6–5.1
HLAB*81	4	P4T,S	1.5 × 10^−5^ ^*a*^	4.0 × 10^−4^	39.8	7.5–211.4	1.3 × 10^−5^ ^*a*^	4.8 × 10^−4^	36.3	7.2–183.0
HLAC*18	4	P4T,S	7.0 × 10^−4^ ^*a*^	9.2 × 10^−3^	12.5	2.9–54.2	5.3 × 10^−3^	9.6 × 10^−2^	17.6	1.6–17.6
HLAB*58	196	G196E,K	9.0 × 10^−4^ ^*a*^	2.2 × 10^−2^	9.1	2.5–33.8	5.9 × 10–^3^	1.8 × 10^−1^	3.8	1.5–9.9

^*a*^ Significant association.

^*b*^ Associations are also shown for the same host alleles when CAPRISA and HIPSS were analyzed jointly (*n* = 134).

Next, we investigated the biological relevance of associations between HLAB*81, HLAC*18, and HLAB*58 with RT. In line with previous studies, we found that HLAB*81 has a predicted binding affinity (% rank 0.04 and IC50 = 561) to a peptide covering RT codons 3–12 (epitope SL10) (Table 2). The predicted binding of HLAB*81 to this epitope suggests that HLAB*81 may have a function in restricting HIV in this region of RT. The predicted binding affinities of other *HLA* alleles were not significant (% rank >1 and IC50 >500).

**TABLE 2:** The sequence and codon number for the SL10 epitope predicted to be restricted by HLAB*81^a^

Epitope	S	P	I	E	T	V	P	V	R	L
Codon	3	**4**	5	6	7	8	9	10	11	12

^*a*^ This sequence covers reverse transcriptase codon 4, where AA variants were significantly associated with HLAB*81.

In addition to RT and PR, *gag* and *nef* sequences were available from the CAPRISA-004 study. *HLA* alleles and chromosome 1 SNPs were tested for association with AA variants in 93 gag and 50 nef codons. Significant associations were observed between rs7519713 and gag codon 18 (*P* = 3.2 × 10^−2^; OR = 4.0, 95% CI = 1.1–14.6) and 147 (epitope ISW9) (*P* = 3.9 × 10^−2^; OR = 3.7, 95% CI = 1.1–12.6) (Table 3). Again, odds ratios show variant AA in gag codons 18 and 147 is observed more in individuals with the minor allele at rs7519713. We did not observe any associations between *HLA* alleles and viral AA variants in gag and nef that passed our significance threshold (Tables S3 and S4). Taken altogether, these findings suggest that the region on chromosome 1, near *CHD1L*, has a function in restricting HIV that is not exclusive to one region of the proteome. Also, the lack of significant *HLA* associations with gag supports the addition of other African cohorts to our data set.

**TABLE 3:** Significant associations between CAPRISA-004 host alleles and viral AA for HIV *gag* (*n* = 49)

Host allele	AA position	AA change	*P* value	FDR	OR	95% CI
rs7519713 (T)	18	K18Q/R	3.2 × 10^−2^	3.9 × 10^−1^	4.0	1.1–14.6
rs7519713 (T)	147	K18L/V/M	3.9 × 10^−2^	3.1 × 10^−1^	3.7	1.1–12.6

### Variability in viral load in HLAB*81 carriers with variant amino acids in reverse transcriptase

**Fig 3:**
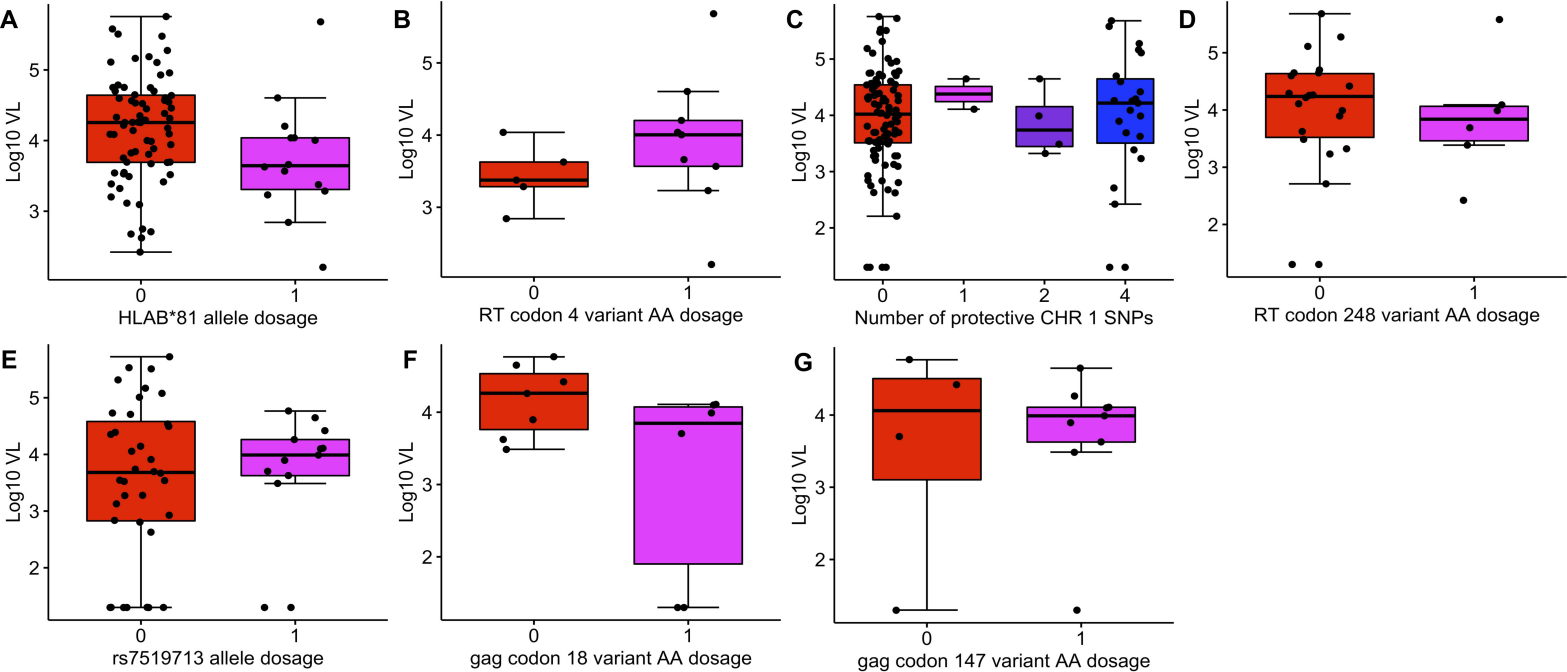
Box plots displaying host allele dosage and AA variant effect on VL. The *y*-axis displays the log-transformed VL (measured as RNA copies/mL of plasma). The *x*-axis displays the allele dosage or variant AA dosage. (**A**) The effect of HLAB*81 allele dosage on VL in HIPSS individuals. (**B**) The effect of a variant AA in RT codon 4 for individuals with the HLAB*81 allele. (**C**) The effect of protective chromosome 1 SNPs on VL in HIPSS and CAPRISA-004 individuals. The *x*-axis displays the number of protective chromosome 1 SNPs the individual has, regardless of homozygosity or heterozygosity. No individuals had three SNPs. (**D**) The effect of variant AA in RT codon 248 on VL for individuals with ≥1 key chromosome 1 SNP. (**E**) The effect of protective chromosome 1 SNP rs7519713 (T) on VL in CAPRISA-004 individuals. (**F**) The effect of variant AA in gag codon 18 on VL for individuals with rs7519713. (**G**) The effect of variant AA in gag codon 147 on VL for individuals with rs7519713. Plot A shows lower VL in the presence of HLAB*81. Plot B suggests a mechanism of viral escape where a variant AA in RT codon 4 counteracts the protective effect of HLAB*81. Plots C and E show little change in VL as a result of chromosome 1 SNPs. Plots D and F show little change in VL as a result of variant AA in gag. AA, amino acid; RT, reverse transcriptase; VL, viral load.

Because of the predicted binding affinity of HLAB*81 with the SL10 epitope, we investigated how the dosage of HLAB*81 and variant AA at RT codon 4 affected VL in the HIPSS cohort. Consistent with previous data (42), we observed that VL was ~0.43 log_10_ copies/mL lower in individuals that had 1 copy of HLAB*81 than those without a copy of the allele (Fig. 3A). When assessing the impact of HIV sequence variation on VL, we found that VL was ~0.48 log_10_ copies/mL higher in HLAB*81 carriers with a variant AA in codon 4 of RT compared to those with the reference allele (Fig. 3B); however, these results were not statistically significant. There was no difference in VL between carriers and non-carriers of HLAC*18 and HLAB*58 (Fig. S1A through C). There was also no difference in VL in carriers that had AA variants in RT codons 4 and 196 (Fig. S1B through D).

In contrast to previous findings (24), we observed no difference in VL in individuals with SNPs rs59784663 (G), rs73004025 (T), rs77029719 (G), and rs7519713 (T) (Fig. 3C and E). There was also no difference in VL in carriers that had AA variants in RT codon 248 and in gag codons 18 and 147 (Fig. 3D, F, and G). In summary, we observed variability in VL due to AA variants in RT codon 4 in carriers of HLAB*81 that warrants further investigation in larger samples.

## DISCUSSION

HIV remains a global health emergency that disproportionately affects individuals in sub-Saharan Africa. Within treated and untreated PLWH, the HIV genome can evolve to escape host pressure to maximize its replication and transmission potential. This evolution leaves traces of host interaction which may be leveraged to uncover host restriction factors or better understand the interplay between host and virus. A previous GWAS identified a locus on chromosome 1 that was associated with control of VL (24). Although the signal was statistically robust and reproducible across multiple cohorts, the underlying biology of what drives the reduction in VL remains unclear. Top associated SNPs were located downstream from the gene *CHD1L*. CHD1L is a helicase and has been classified as an SNF2-like protein, with diverse functions in DNA transcription and repair (43, 44). CHD1L is activated by poly(ADP-ribose), is recruited to sites of DNA damage by PARP-1, and has functions in DNA repair through chromatin relaxation (43 – 45). In addition, TRIM33 is dependently recruited to sites of DNA damage by PARP-1 and CHD1L, where it acts as a transcriptional suppressor (46, 47). The helicase-like activity of CHD1L and its interaction with PARP-1 and TRIM33 could impact HIV integration, transcription, and the production of viral proteins. We hypothesized that by testing for association between HIV and host variability, we could identify specific HIV genes targeted by CHD1L, therefore shedding light on how it might restrict HIV replication.

We identified several associations between chromosome 1 SNPs and AA variants in RT and gag. Specifically, rs59784663, rs73004025, rs7519713, and rs77029719 were associated with variants in RT codon 248. Chromosome 1 SNP rs7519713 was also significantly associated with variant AA in gag codons 18 and 147 (epitope ISW9). The chromosome 1 SNPs included in the present study have only recently been associated with variance in VL (24) and for the first time have been associated with variant AAs in RT and gag. These findings support the hypothesis that CHD1L has a function in restricting HIV. However, our study was limited in power by small sample size, and therefore, we suggest further studies to fully understand the mechanism of restriction.

Significant associations were also seen between class I two-digit resolution *HLA* alleles and AA variants in RT. In the HIPSS cohort, HLAB*81 and HLAC*18 were found to be significantly associated with variants in RT codon 4 and HLAB*58 with RT codon 196. The association of HLAB*81 and RT codon 4 (epitope SL10) has been previously described (19, 48 – 50). The association of HLAC*18 with RT codon 4 (epitope SL10) has not been previously shown. A study done by Smith et al. identified an elite controller of HIV with both HLAB*81 and HLAC*18 alleles (51). HLAB*81 and HLAC*18 were not found to be in linkage disequilibrium within our cohort, so it is unlikely that the association of HLAC*18 with SL10 is due to HLAB*81. RT codon 196 has been previously associated with other *HLA* alleles (50) but not with HLAB*58. HLAB*81 and HLAB*58 have been previously implicated with reduced viral loads, higher CD4^+^ T-cell counts, and mutations in HIV; however, this has primarily been investigated in the *gag* and *nef* genes (18, 22, 52 – 54). Significant associations between host alleles and RT were not replicated in the CAPRISA-004 cohort. Quality control filtering of HIPSS and CAPRISA-004 genotype data was done separately, and the small sample size (*n* = 62) resulted in some alleles having MAFs below filter thresholds and were therefore not included in the analysis. Due to the selection threshold, well-documented escape mutations could not be tested; for example, the HLAB*57:03/57:02/58:01 association with the gag codons 240–249 (TW10 epitope), HLAB*57:03 with gag codons 162–172 (KF11 epitope), and HLAB*81:01 with gag codons 180–188 (TL9 epitope).

We further investigated associations between *HLA* alleles and viral epitopes. Overall, we found that HLAB*81 had a predicted binding affinity for an epitope which covered RT codon 4 (epitope SL10) (% rank 0.04 and IC50 = 561). The predicted binding affinity of HLAB*81 suggests that it may have a function in restricting HIV in that region of RT. However, based on a review of the IEDB experimental catalog (55) and the Los Alamos HIV Molecular Immunology Database (56), the RT epitope has not yet been experimentally confirmed as a major histocompatibility complex (MHC) ligand.

Individuals carrying the HLAB*81 allele had lower VLs than those without a copy of the allele. In addition, VL was higher in HLAB*81 carriers with a variant AA in codon 4 of RT (epitope SL10), although these results did not reach statistical significance. The predicted binding affinity of HLAB*81 and higher VLs in individuals with variant AA in RT codon 4 highlights a region of immune pressure on the viral genome. A previous study (49) found that P4T/S in epitope SL10 reduces viral fitness resulting in reversion of the AA variant. Similarly, we found that in the absence of HLAB*81, the mean log_10_ VL of P4T/S was lower than the mean log_10_ VL of those with a consensus AA (Fig. S1E). In addition, Leitman et al. detected the P4T/S variant ~4 months after mother-to-child transmission of HIV, where the mother was HLAB*81 ^−/−^ and the child was HLAB*81 ^+/−^ (19). Together with our findings, these data support that HLAB*81 is driving the P4T/S mutation we see in our study. HLAB*81 has also been shown to restrict HIV p24 gag*,* and HIV responds with a T186S mutation in the restricted epitope (18, 22, 57). T186S reduces the replicative capacity of HIV (18, 57), which is similar to the trend in RT codon 4, where in the absence of HLAB*81, the mean log_10_ VL is lower in individuals with P4T/S (Fig. 3B and Fig. S1E). T186S compensatory mutations in gag provide little improvement to viral replicative capacity (18) and VL, whereas the role of compensatory mutations for RT codon 4 is not yet described.

The HIPSS and CAPRISA-004 cohorts were chosen for this study because they were treatment naïve and represent an African population at high risk of HIV infection (58). This presents a unique opportunity to study HIV co-evolution with available human and HIV sequence data. Genomes of individuals vary greatly by their ancestral history, and individuals of African ancestry have the most diverse genomes with low linkage disequilibrium, the most ancestral specific SNPs, and number of rare SNPs (58, 59). In genomic studies, large cohort sizes are needed in genetically diverse populations to identify significant SNPs with minor effects. For that reason, this study had several limitations. Primarily, the small sample size and limited availability of sequence data prevented a full genome-to-genome analysis and had limited power to detect associations with modest effect sizes. Thus, we restricted our analysis to loci previously reported to impact HIV replication. In some cases, this also limited our ability to test previously reported associations. For example, previously described gag associations with HLAB*81 could not be tested due to low allele frequency (<0.05) in the CAPRISA-004 cohort. However, it is important to note that CAPRISA-004 participants were in early stages of their infection, while the HIPSS cohort would have had participants at different infection stages. Therefore, the HIPSS cohort likely better represents a treatment-naïve population during chronic infection, both in the case of viral sequences and VL. This could serve as another explanation for why HIPSS associations were not replicated in the CAPRISA-004 cohort. Our sample size provided ~80% power to detect an HIV AA change in 5% of individuals with an OR of >4 at *P* < 0.05. While our sample size is small, we were able to replicate the association between HLAB*81 and RT codon 4, suggesting our approach is valid in principle.

Two HIV genome-to-genome analyses have been previously conducted with cohorts of >1,000 individuals of European (14) and mixed ancestries (60). Both studies found that genome-to-genome analyses produced stronger associations than host (14) or viral (60) genome-to-VL analyses. A similar trend was seen in the present study, where significant associations were only seen when analyzing associations between host and viral variants (Table 1). Therefore, we feel our data warrants future studies in additional cohorts to fully assess the impact of host genetic variability near *CHD1L* on HIV evolution in African populations.

### Conclusion

HIV remains a major public health concern which disproportionately affects low-income countries and is concentrated in Southern and Eastern Africa. Controlling VL in PLWH remains the key goal to end the pandemic. The present study demonstrated that HIV sequence variation can be used as a phenotype in host genome studies of African populations. In addition, this study found that protective SNPs on chromosome 1 are significantly associated with AA variants in *RT* and *gag* genes. However, the present study was limited by a small cohort size, which prevented the inclusion of more viral and host alleles in analyses. Moving forward, it is imperative that similar analyses are conducted in larger populations in order to address the burden of HIV in Africa and to better understand host control of HIV in these populations.

## SUPPLEMENTAL MATERIAL

Fig. S1 jvi.00954-23-s0001.pdf Supplemental figure.

Tables S1 to S6 jvi.00954-23-s0002.xlsx Supplemental tables.

ASM does not own the copyrights to Supplemental Material that may be linked to, or accessed through, an article. The authors have granted ASM a non-exclusive, world-wide license to publish the Supplemental Material files. Please contact the corresponding author directly for reuse.
