# Supplementary material for: Host genetic variation at a locus near CHD1L impacts HIV sequence diversity in a South African population
Source: J Virol. 2023 Sep 25;97(10):e00954-23. doi: 10.1128/jvi.00954-23 (PMC10617395; doi:10.1128/jvi.00954-23)
Supplement: Fig. S1 — Supplemental figure. [file jvi.00954-23-s0001.pdf]

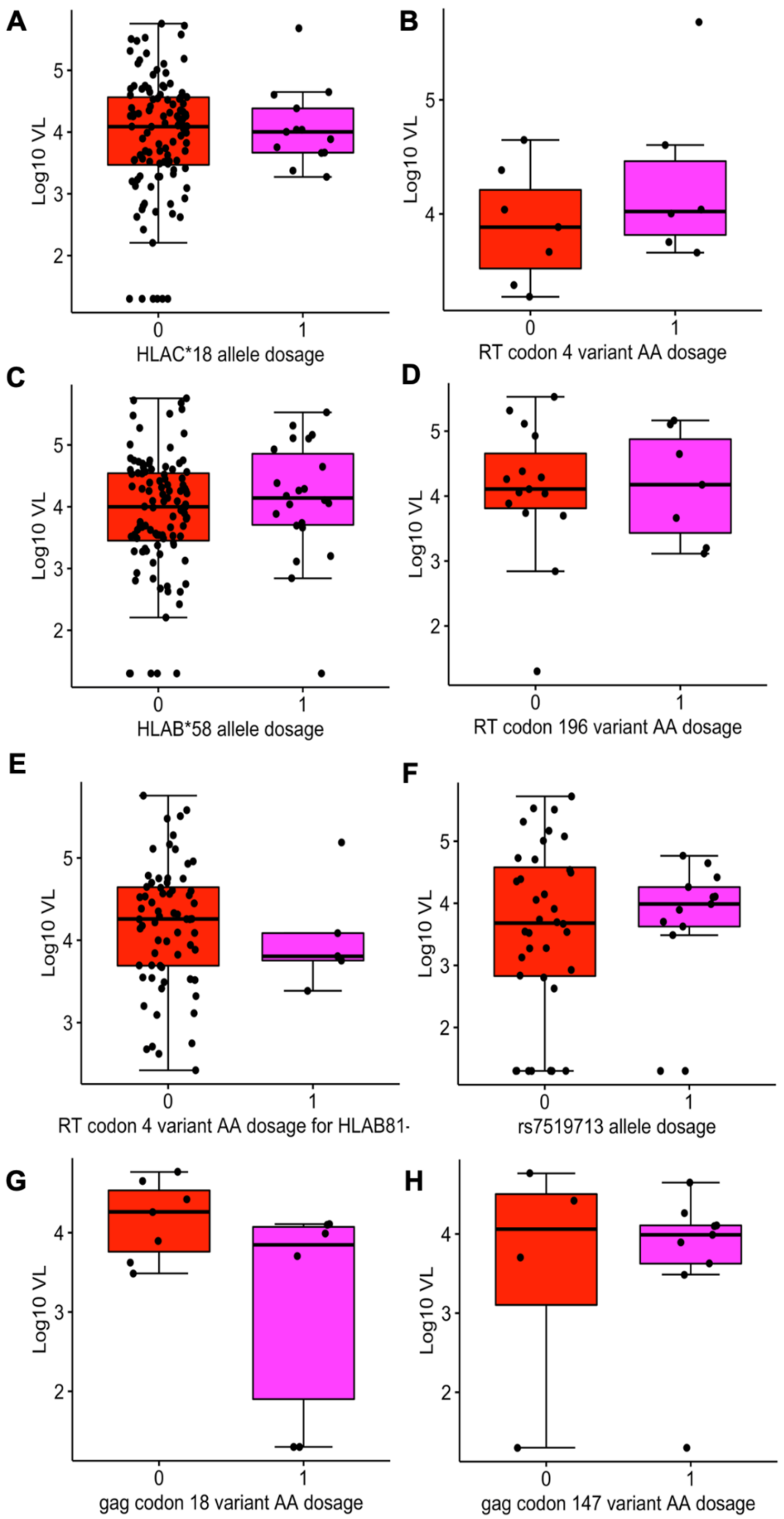

Figure S1. Box plots displaying host allele dosage and AA variant effect on VL. The Y-axis displays the log transformed VL (measured as RNA copies/mL of plasma). The X-axis displays the allele dosage or variant AA dosage. (A) The effect of *HLAC*18* allele dosage on VL in HIPSS and CAPRISA-004 individuals. (B) The effect of a variant AA in RT codon 4 for individuals with the *HLAC*18* allele. (C) The effect of *HLAB*58* allele dosage on VL in HIPSS and CAPRISA-004 individuals. (D) The effect of variant AA in RT codon 196 on VL for individuals with the *HLAB*58* allele. (E) A boxplot showing the effect of a variant AA in RT codon 4 for HIPSS individuals without the *HLAB*81* allele. (F) The effect of rs7519713 allele dosage on VL in CAPRISA-004 individuals. (G) The effect of a variant AA in gag codon 18 for individuals with rs7519713. (G) The effect of a variant AA in gag codon 147 for individuals with rs7519713. Plots A and C show little change in VL as a result of these *HLA* alleles. Plots B and D show little change in VL as a result of variant AA in RT. Plot E shows that a variant AA in RT codon 4, in the absence of *HLAB*81*, results in lower VL. Plot F shows little change in VL in the presence of rs7519713. Plots G and H show there little change in VL as a result of variant AA in gag in individuals with rs7519713.

RT, reverse transcriptase; AA, amino acid; VL, viral load.
